# 3D cephalometric landmark detection by multiple stage deep reinforcement learning

**DOI:** 10.1038/s41598-021-97116-7

**Published:** 2021-09-01

**Authors:** Sung Ho Kang, Kiwan Jeon, Sang-Hoon Kang, Sang-Hwy Lee

**Affiliations:** 1 Division of Medical Mathematics, National Institute of Mathematical Science, Daejeon, Republic of Korea; 2 Department of Oral and Maxillofacial Surgery, National Health Insurance Service Ilsan Hospital, Goyang, Republic of Korea; 3 Department of Oral and Maxillofacial Surgery, Oral Science Research Center, College of Dentistry, Yonsei University, Seoul, Republic of Korea

**Keywords:** Machine learning, Digital radiography in dentistry

## Abstract

The lengthy time needed for manual landmarking has delayed the widespread adoption of three-dimensional (3D) cephalometry. We here propose an automatic 3D cephalometric annotation system based on multi-stage deep reinforcement learning (DRL) and volume-rendered imaging. This system considers geometrical characteristics of landmarks and simulates the sequential decision process underlying human professional landmarking patterns. It consists mainly of constructing an appropriate two-dimensional cutaway or 3D model view, then implementing single-stage DRL with gradient-based boundary estimation or multi-stage DRL to dictate the 3D coordinates of target landmarks. This system clearly shows sufficient detection accuracy and stability for direct clinical applications, with a low level of detection error and low inter-individual variation (1.96 ± 0.78 mm). Our system, moreover, requires no additional steps of segmentation and 3D mesh-object construction for landmark detection. We believe these system features will enable fast-track cephalometric analysis and planning and expect it to achieve greater accuracy as larger CT datasets become available for training and testing.

## Introduction

Cephalometry using three-dimensional (3D) computerized tomography (CT) images for craniofacial morphometry has been applied in various medical and biological fields^1^. Two-dimensional (2D) cephalometry has long played a central role in such applications. Recent scientific and technological developments have prompted the rapid introduction of 3D cephalometry due to its advantages with respect to accurate anatomical identification and complex structural evaluation. Despite these remarkable advantages, the considerable time and expertise needed for manual landmarking on 3D data has posed a major obstacle to widespread adoption of 3D cephalometry.

Various machine learning algorithms for 3D automatic cephalometric landmark detection have recently yielded striking results^2–5^, especially compared with the model- or knowledge-based approaches^6–8^. In a recent review of 3D cephalometric landmarking^5^, deep learning^2,4^ was noted to perform better than other methods. Deep learning methods using convolutional neural networks, however, predict a spatial location by a single-shot decision based on training results from huge amounts of labelled data. This decision-making process cannot be properly adapted to complex structures with variation/deformation. On the other hand, deep reinforcement learning (DRL) performs prediction through sequential dynamic interaction with the environment, an approach frequently ignored when implementing of deep learning in the medical field^9^.

Through the clinical performance of cephalometric analysis^10–12^ as well as our own evaluation of 3D cephalometric studies, we realized that professional landmarking by experts/doctors tends to share a common pattern: the operator’s attention first focuses on the global features of the image, based on anatomical knowledge and the characteristic orientation of the radiographical image. It then moves to the local region of interest to catch the local features for final annotation of the landmark coordinate values. This pattern of global to local attention shift is well known and has been applied to automatic cephalometry, particularly 2D cephalometry^13–16^.

**Figure 1:**
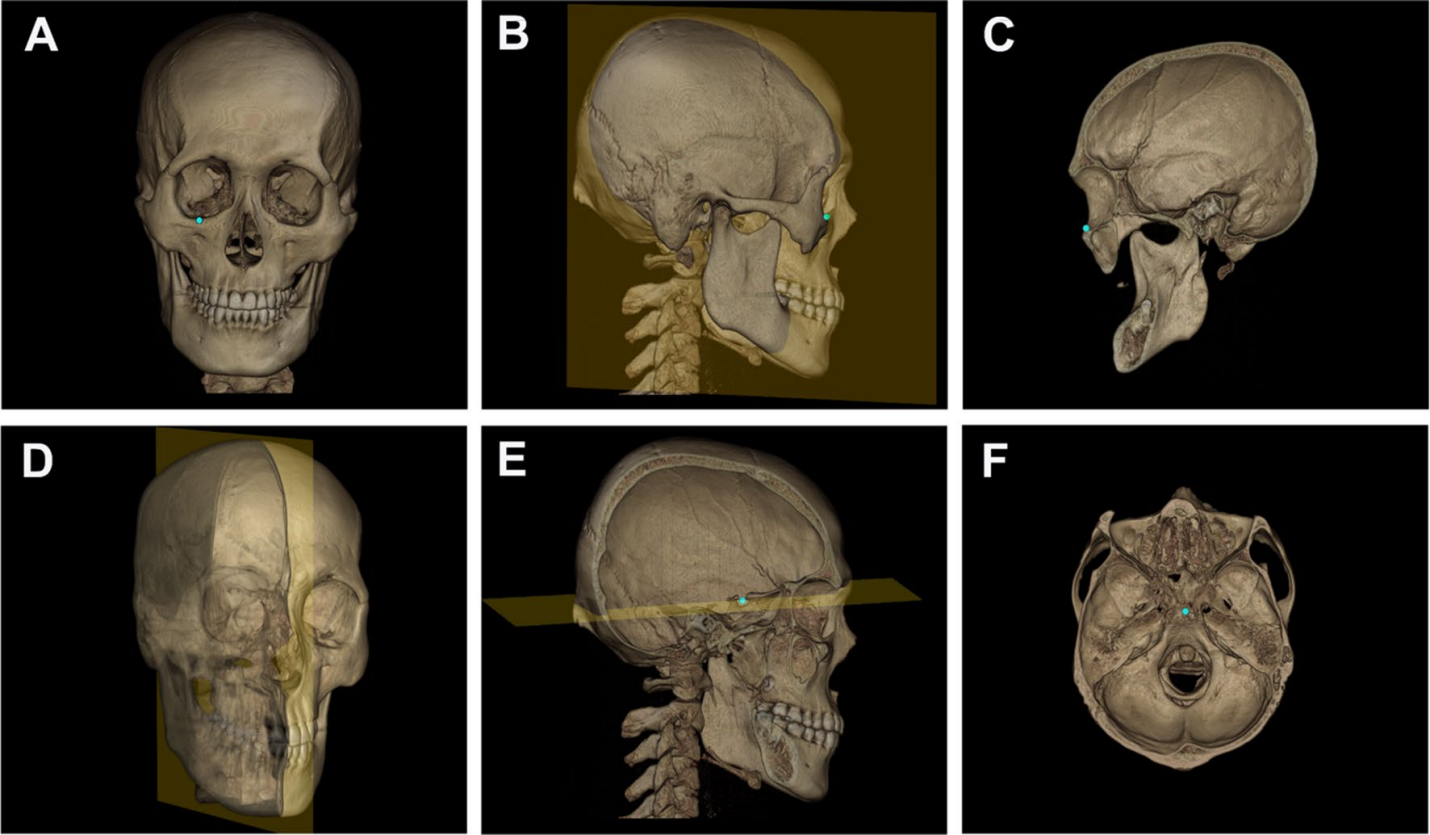
The sequential selection of observational views in multiple stages for 3D landmark pointing, considering the anatomical characteristics of landmarks. Blue points indicate reference points determined by clinical experts. (**A**–**C**) a multiple-staged directional sequence for right orbitale. (**A**) Anterior view, (**B**) right lateral view, and (**C**) sagittal cut left lateral view. (**D**–**F**) a multiple directional sequence for sella point. (**D**) Semi lateral 3D view with transparent half skull. (**E**) Sagittal cut right view, and (**F**) axial cut top view.

However, 3D landmark annotation of a 3D model cannot be simply completed using this approach due to the increase in complexity and dimension of anatomical structures compared to those in 2D. Experts generally observe the 3D model first move to the region of interest, then tentatively determine the landmark position on the 3D model, finally confirming and adjusting the landmark on the appropriately selected sectional or cutaway view. Figure 1 shows sequential selection of observational views in multiple stages for orbitale (Fig. 1A–C) and sella (Fig. 1D–F), considering the anatomical characteristics of each landmark. We thus recognize that professional landmarking involves a sequential multi-stage procedure based on the morphological characteristics and global–local feature of landmarks.

We therefore assumed that the spatial localization task in 3D cephalometric landmark detection can be formulated as a Markov decision process, which is a sequential decision-making process in a stochastic environment^17^. We considered DRL, known for being a model-free algorithm, for solving this 3D localization issue and hypothesized that we could let the DRL agent learn the optimal navigation paths in the representation of 2D projected images from 3D volume data for cephalometric landmarking. DRL is also useful in working with limited labeled data, as frequently occurs in medical research involving normal or diseased human model. Based on our search of the literature, we believe this is the first study to apply DRL to the field of 3D automatic cephalometry.

Cephalometric landmarks have different anatomical or geometrical characteristics, being located on a 3D surface, in 3D space, or within the bone cavity. We thus surmised that the application of DRL in one, two, and three stages might be differentially effective depending on landmark characteristics. We therefore constructed a staged DRL landmark detection system and evaluated the accuracy level of the different DRL stagings for its justification.

**Figure 2:**
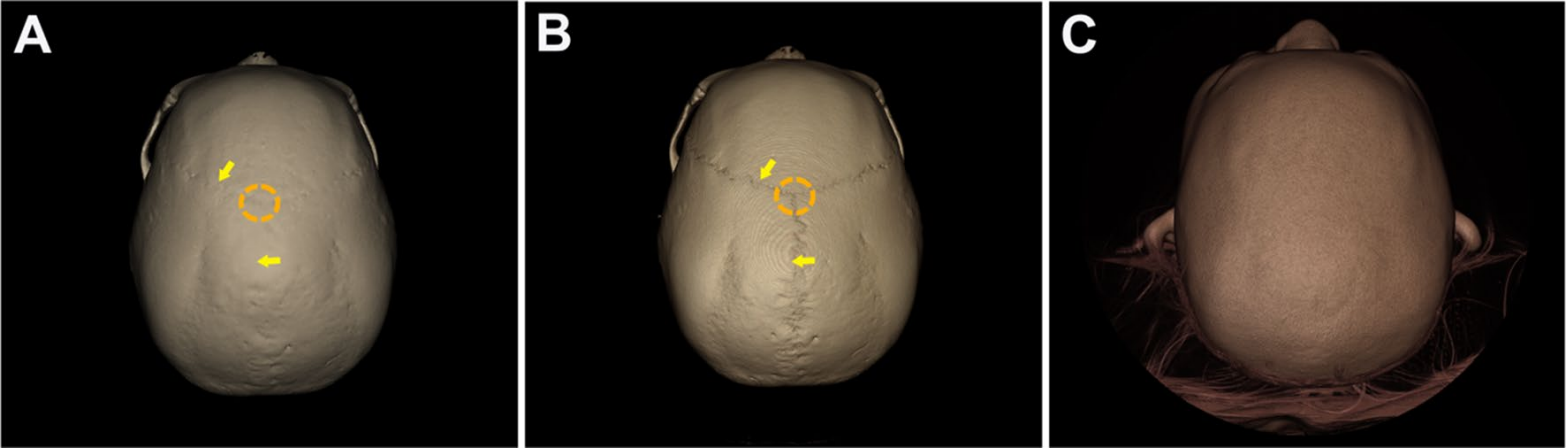
Different modeling images for the same cranial vault seen from the top view of the same subject. (**A**) 3D mesh-model in stereolithographic format with faint coronal suture and bregma, but invisible sagittal suture; (**B**) another top view of the same cranial vault by volume-rendered modeling in bone setting, showing these structures more clearly; (**C**) the same top view of skull, produced by volume rendering in a soft tissue setting, showing the nose, ear, and other skin morphology.

For the optimal visualization of 3D objects on CT images, we here utilized volume rendering, rather than 3D triangular mesh- or polygon mesh-modeling. Volume rendering is a well-known technique for visualizing a 3D spatial dimension of a sampled function by calculating a 2D projection of a color-translucent volume^18^. Figure 2 compares three top views of the same cranial vault produced from the same CT data of a subject: a stereolithographic mesh-modeled skeletal image (Fig. 2A), and a volume-rendered skeletal (Fig. 2B), and a soft-tissue image (Fig. 2C). The landmark bregma (shown at the center of the dotted circle line in Fig. 2A,B) and adjacent coronal and sagittal suture (marked by arrows) of the skull are comparatively visualized. The landmark and structures can be observed more clearly on the volume-rendered view (Fig. 2B) than those in the mesh-modeled view (Fig. 2A).

The objective of this study was to develop an automatic 3D cephalometric annotation system using volume-rendered imaging and selective single- or multi-stage DRL, based on professional human landmarking patterns and characteristics of landmarks. The accuracy was confirmed by comparing the results of our DRL system with those of human experts, then correlated with the type of landmarks or DRL application stages. We found that multi-stage DRL performed well in achieving statistically significant improvement in the accuracy level of detected landmarks.

The main contributions of the proposed method are summarized as follows:

- first study to apply DRL in multi-stages to 3D automatic landmark detection
- characterization of multi-staged DRL annotation strategy based on stage-dependent accuracy level and anatomical characteristics of landmarks
- a simpler procedure which avoids segmentation by applying volume rendering, crucially supporting practical application
- consistent detection accuracy of landmarks, regardless of their types, based on a multi-stage approach and mimicking human landmarking patterns

The remainder of this paper presents a review of the literature on automatic 3D cephalometric landmark detection, describes our methods and materials, sets out the experimental results, then closes with a discussion and conclusion.

## Related works

### Classical machine learning-based approach

Knowledge-based approaches utilize mathematical anatomical or geometrical descriptions of landmarks and surrounding structures^8,19,20^. Even though such approaches reflect human landmarking, the edge detection of 3D anatomical contours or the landmark localization onto an edge or contour are difficult to perform. The model (atlas)-based approach aims to register a referenced mathematical or statistical model with landmarks on the test model and to transfer those landmarks^6,7,15,21^. A properly produced model can be well-matched with the test model, but this might not be appropriately customized to variation or deformation of the complex craniofacial structure. Generally, greater levels of inaccuracy are reported at the level of 2.4–3.4 mm^6,7^, while Ridel et al.^21^ recently reported a mean error of 1.64 mm on hard tissue. Both of these approaches were unable to achieve robust landmarking due to the great variations in size, shape, or position of the structure, which are frequently found in medical images. This limitation can be addressed with learning-based approaches, which are trained on sampled images with great geometrical variation.

### Deep learning-based approach

Recent automatic 3D landmarking studies have mainly utilized the deep learning-based approach^2–4^, which can properly address ambiguity in landmarking of the complex craniofacial structure by virtue of its enhanced efficiency, adaptation capability, and low sensitivity to noise. However, large training datasets are needed to overcome anatomical variation. Zhang et al.^2^ and Lee et al.^22^ reported good mean errors of less than 1.5 mm using deep learning, while obtaining a limited number of landmarks due to memory constraints^2^ or lack of expansibility^22^. Though this approach shows an improved capability to recognize landmarks in medical images, it struggles to handle high dimensional image data and requires large training datasets to interpret models with anatomical variation. In addition, 3D landmarking requires a sequential process, as do many other medical decision-making procedures^9^. The one-shot decision process of deep learning has difficulty in handling this localization issue.

### DRL-based approach

DRL has recently drawn attention due to its capability in 3D localization^23,24^. It learns the optimal path by maximizing the accumulated rewards of sequential action steps. Ghesu et al. first applied DRL to 3D landmark detection in fixed- or multi-scale models to obtain detection accuracy of 3 mm or less for skeletal or soft tissue^23,24^. Alansary et al. reported several different deep Q-network (DQN)-based models for 3D landmark detection in magnetic resonance and ultrasound images, finding the models outperformed the previous study results^25^. Despite considerable promising research, the models were not applied to 3D cephalometric landmark detection.

## Methods

### Subjects and CT data

CT data from our previous 3D cephalometric study of normal subjects were used^26^. Twenty-eight normal Korean adults with skeletal class I occlusion volunteered, informed consent being obtained from each subject. The work was approved by the Local Ethics Committee of the Dental College Hospital, Yonsei University, Seoul, Korea (IRB number: 2-2009-0026). All methods were carried out in accordance with relevant guidelines and regulations in the manuscript. Both clinical and radiographic examinations were used to rule out facial dysmorphosis, malocclusion, or history of surgical or dental treatment. The subjects were anonymized and divided into two groups, the training group (n = 20) and the test group (n = 8).

### Landmarks

**Figure 3:**
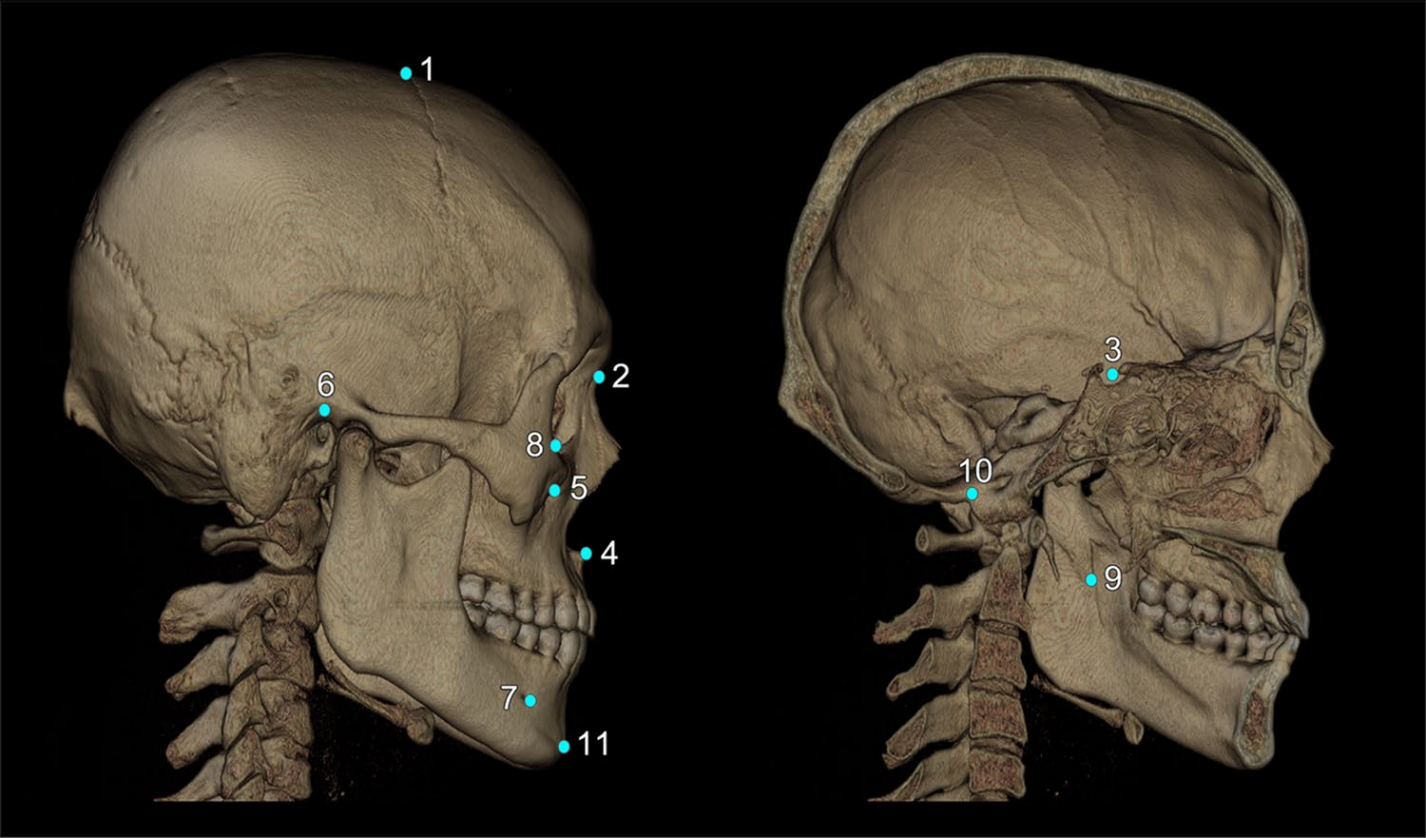
3D cephalometric landmarks used in this study were marked on the skull, some of them on the surface and others inside the skull; others were in the confined space. The total number of landmarks was 16, five being bilateral. All landmarks are defined and explained in Supplementary Table 1. They are also classified into three landmark types according to Bookstein’s landmark classification, as seen below in notes 2–4. *Note* (1) the name of landmarks used in this study: 1. bregma; 2. nasion; 3. sella; 4. anterior nasal spine (ANS); 5. infraorbital foramen (IOF, bilateral); 6. porion (bilateral); 7. mental foramen (MF, bilateral); 8. orbitale (bilateral); 9. mandibular foramen (F, bilateral); 10. center of foramen magnum (CFM); 11. Pogonion. *Note* (2) Type 1 landmarks (n = 2): bregma, nasion. *Note* (3) Type 2 landmarks (n = 8): sella, anterior nasal spine, infraorbital foramen, orbitale, mental foramen. *Note* (4) Type 3 landmarks (n = 6); porion, center of foramen magnum, mandibular foramen, pogonion.

The following craniofacial and mandibular cephalometric landmarks (total N = 16) were included in this study (Fig. 3): bregma, nasion, center of foramen magnum, sella turcica, anterior nasal spine, pogonion, orbitale, porion, infraorbital foramen, mandibular foramen, and mental foramen. The latter five points were bilateral, and the others unilateral. These points are applicable to general cephalometric analysis, but may not be sufficient for a specific analysis, such as Delaire’s^26^. Each landmark’s definition, position, and type is described in Fig. 3 and Supplementary Table 1.

Two experts, each having done 3D cephalometry for more than 10 years in a university hospital setting, independently located these 16 landmarks for 3D cephalometric analysis with Simplant software (Materialise Dental, Leuven, Belgium)^27^. Their mean landmark coordinate values were used as the standard to evaluate DRL prediction accuracy in this study. The coordinate value on the $x$-axis indicated the transverse dimension, the $y$-axis the anterior–posterior dimension and the $z$-axis the top–bottom dimension. The coordinate value of each landmark in Simplant software was exported in Digital Imaging and Communications in Medicine (DICOM) format to construct the label data using the StoA software (Korea Copyright Commission No. C-2019-032537; National Institute for Mathematical Sciences, Daejeon, Korea).

The landmarks have different characteristics which can be classified into three types based on their structural location and informed by biological processes and epigenetic factors^28,29^. Although landmark typing is not highly consistent across several studies^29^, we classified them into three types^28,29^, as follows: the type 1 landmark (on discrete juxtaposition of tissues), including bregma and nasion; the type 2 point (on maxima of curvature or local morphogenetic processes), involving sella, anterior nasal spine, infraorbital foramen, orbitale, and mental foramen; finally, type 3 points, comprising porion, center of foramen magnum, mandibular foramen, and pogonion.

### General scheme

**Figure 4:**
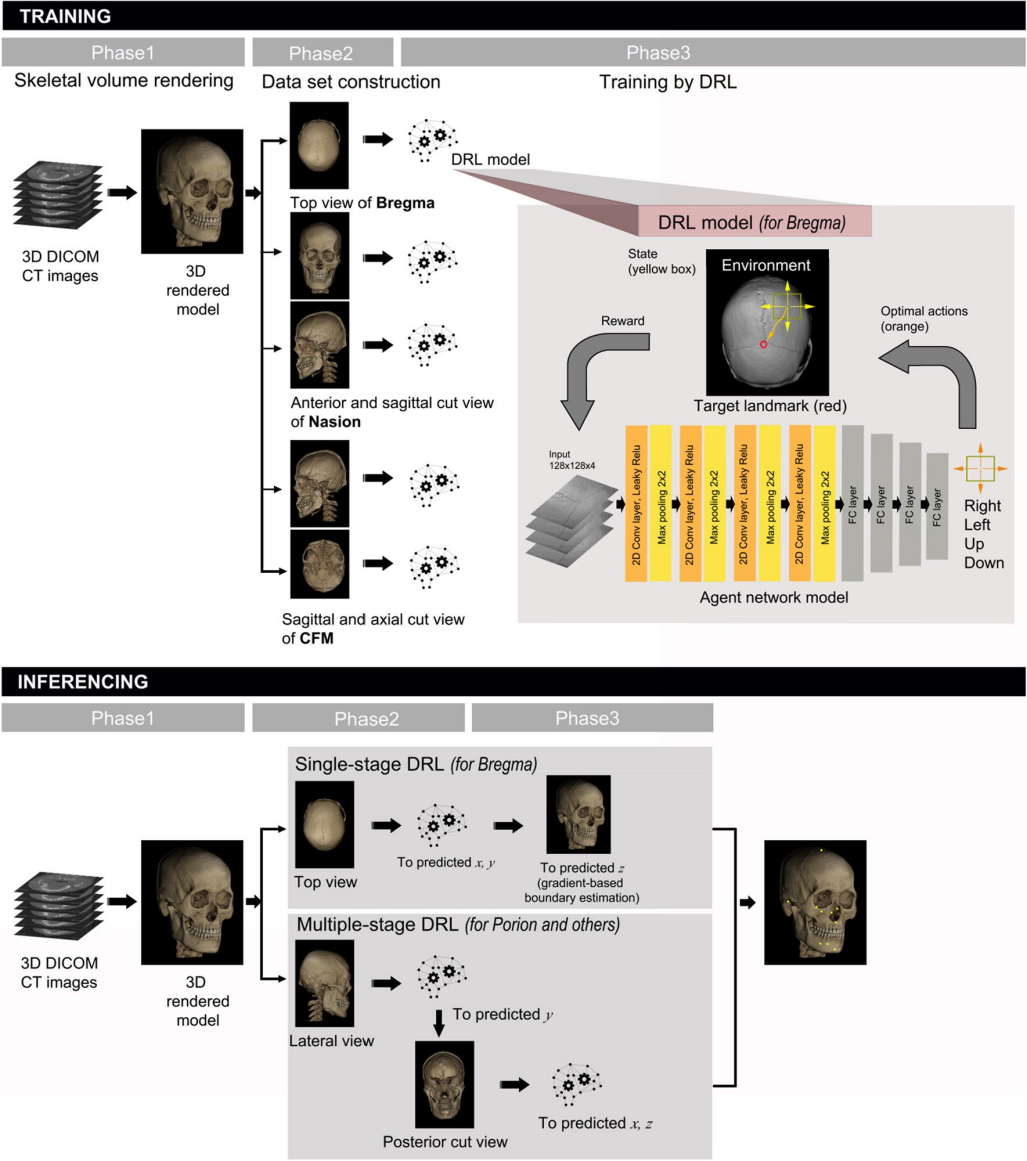
Schematic diagram of the proposed 3D cephalometric landmark detection framework using deep reinforcement learning (DRL). The training stage included data import and volume rendering in the first phase, anatomical image view adjustment for landmarks in the second phase, and training with DRL agents in the third phase. The latter is depicted in detail in the upper box with a drawing to illustrate the agent navigating toward the target landmark. The inferencing stage included both single- and multi-stage DRL, starting with the same 3D model rendering in phase 1, followed by single-stage or multi-stage DRL in the second phase, and finalized by gradient-based boundary estimation for single-stage DRL, or by repeated DRL and landmark prediction for multi-stage DRL in the third phase.

CT data in DICOM format were transferred to a personal computer and a volume rendered 3D model was produced by the following steps: a 2D-projection image was acquired by ray-casting, and the transparency transfer function was applied for bone setting (as shown in the first phase of training in Fig. 4). To compose the dataset, we adjusted the image for each landmark by anatomical view in gray and to $512\times 512$ in pixel size. The adopted main views were top, bottom, anterior, posterior, or lateral (right or left) view of the 3D model and their cutaway views (defined as a 3D graphic view or drawing in which surface elements of the 3D model are selectively removed to make internal features visible without sacrificing the outer context entirely), as shown in Fig. 1C,E,F. To locate the landmark on cutaway view, voxel pre-processing was performed with transparency application in the region of no interest. A dataset was constructed by combining the obtained images and labelled landmark with its pixel location in the corresponding image view domain, which had been converted from DICOM coordinates to image pixel coordinate.

Single or multiple views were appropriately produced for each landmark and DRL training was performed without data augmentation on the 20 training group models (as shown in the second and third phases of the training stage in Fig. 4). DRL training was organized in such a way that the environment responds to the agent’s action and the agent continuously acts to get maximum rewards from the environment^17^, i.e., to reach the closest location to the reference coordinate (as shown in the third phase of training in Fig. 4).

At the inferencing stage, landmark prediction was performed by both single-stage and multi-stage DRL to evaluate the landmark- or stage-related accuracy level (as shown in the first phase of inference stage in Fig. 4). Single-stage DRL refers to one pass of the DRL algorithm, followed by gradient-based boundary estimation, for landmark detection. The multi-stage DRL was defined as the application of a single-stage DRL algorithm for more than two passes, without the gradient-based boundary estimation. The single-stage DRL was not applicable to landmarks located in 3D empty space, such as foramen magnum or sella. These landmarks therefore needed to be determined by multi-stage DRL, whereas other points could be inferenced by both single- and multi-stage DRL (as shown in the second and third phase of the inferencing stage in Fig. 4).

### DRL for cephalometric landmark detection

The DRL training framework known as Double DQN^30^ was adopted after comparing its performance with that of other DQNs for 3D landmarking. DQN handles unstable learning and sequential sample correlation by applying the experience replay buffer and the target network, achieving human-level performance^31^. Double DQN achieves more stable learning by utilizing the DQN solution to the bias problem of maximum expected value^30^.

The DRL agent learns the optimal path trajectory to a labeled target position through a sequential decision process. We formulated the cephalometric landmark detection problem as a Markov decision process, defined by $\mathcal{M}=(\mathcal{S},\mathcal{A},\mathcal{P},\mathcal{R})$ where $\mathcal{S}$ is the set of states, $\mathcal{A}$ set of actions, $\mathcal{P}$ the state transition probabilities, and $\mathcal{R}$ the reward function. In this study, environment $E$ is an image ($512\times 512$) obtained through volume rendering from DICOM data with ground truth landmark position. The agent’s action $a\in \mathcal{A}$ was defined as movements on the 2D image plane (right, left, up, and down), along the orthogonal axis in an environment image. The state $s\in \mathcal{S}$ was defined as a region of interest image from the environment wherein the agent was located. It was zoomed to various pixel resolutions with a fixed pixel size of $128\times 128$. The reward function ${\mathcal{R}}_{t}$ was defined by the Euclidean distance between the previous and current agents at time $t$ as follows:

1 $${\mathcal{R}}_{t}=Dist \left({AP}_{t-1}, TP\right)-Dist ({AP}_{t}, TP)$$

where $AP$ represents the predicted image position on the image of given $E$, and $TP$ is the target ground truth position. The agent receives a reward from the environment after valid action in every step. The state action function $Q(s,a)$ is then defined as the expectation of cumulative reward in the future with discount factor $\gamma$. More precisely,

2 $$Q\left(s,a\right)={\mathbb{E}}\left[{\mathcal{R}}_{t}+\gamma {\mathcal{R}}_{t+1}+{\gamma }^{2}{\mathcal{R}}_{t+2}+\cdots +{\gamma }^{n-1}{\mathcal{R}}_{t+n-1 }| s,a\right]$$

Using the Bellman optimality equation^32^, the optimal state action function ${Q}^{*}(s,a)$ for obtaining the optimal action is computed as the following:

3 $${Q}^{*}\left(s,a\right)={\mathbb{E}}\left[{\mathcal{R}}_{t+1}+\gamma \underset{{a}^{^{\prime}}}{\mathrm{max}}{Q}^{*}({s}_{t+1}, {a}^{^{\prime}})| s,a\right]$$

Q-learning finds the optimal action-selection policy by solving Eq. (3) iteratively^33^. Due to the heavy computation needed, an approximation by the deep neural network $Q\left(s,a;\theta \right)$ was adopted instead of $Q\left(s,a\right)$, where $\theta$ is the parameter of the deep neural network. Double DQN algorithm minimizes the function $L(\theta )$ as defined by the following:

4 $$L\left(\theta \right)={\mathbb{E}}\left[{\left({\mathcal{R}}_{t+1}+\gamma Q\left({s}_{t+1}, \underset{a}{\mathrm{argmax}}Q({s}_{t+1},a;\theta ){;\theta }^{-}\right)-Q({s}_{t}, a;\theta )\right)}^{2}\right]$$

where ${\theta }^{-}$ represents the frozen target network parameters. The target network ${Q(\theta }^{-})$ was periodically updated by parameter values copied from the training network $Q\left(\theta \right)$ at every $C$ step. The update frequency $C$ of the target network was empirically set to check the convergence of the loss function. Gradient clipping was applied to limit the value within $[-1, 1]$, as suggested by Mnih et al.^31^. To avoid the sequential sample correlation problem, experience replay buffers (denoted by $D$) were used, consisting of multi-scale resolution patch images ($128\times 128$) extracted by the agent’s action in our training process. Random sampling tuples $[{s}_{t}, {a}_{t}, {r}_{t}, {s}_{t+1}]$ were configured in batches and trained^31^. Details of training steps for our DRL are described in Algorithm 1 (in pseudocode) and Fig. 4. A multi-scale agent strategy was used in a coarse-to-fine resolution manner^23–25^. Our termination condition was set to the case of fine resolution and the most duplicate agent position in the inferencing phase.
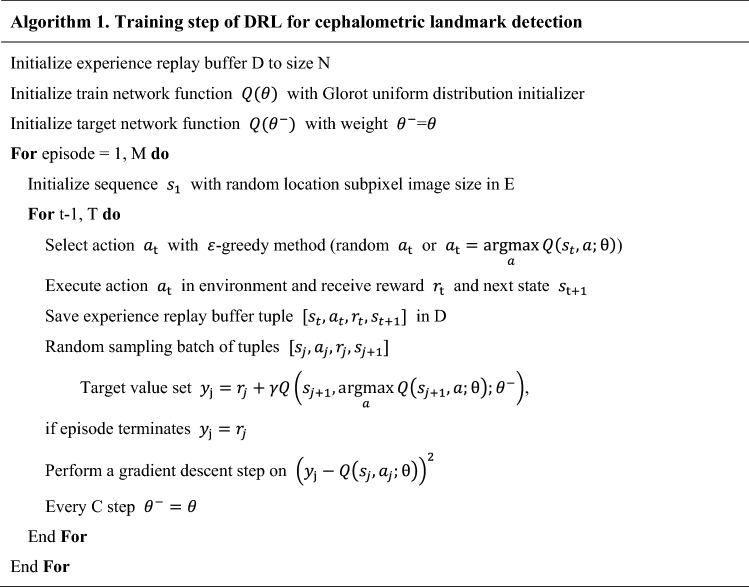


The agent network contained four convolutional layers with $128\times 128\times 4$ as input (frame history $k$ is 4), each followed by a leaky rectified linear unit, and four $2\times 2$ max pooling with stride 2 for down-sampling. The first and second convolutional layers convolved 32 filters of $5\times5$. The third and fourth convolutional layers convolved 64 filters of $3\times 3$. All convolutional layers’ stride was 1. The last pooling layer was followed by four fully-connected layers and consisted of 512, 256, 128, and 4 rectifier units, respectively. The final fully-connected layer had four outputs with linear activation. All layer parameters were initialized according to the Glorot uniform distribution. Figure 4 illustrates the agent network model in the third phase of the training stage.

### Single-stage DRL

**Figure 5:**
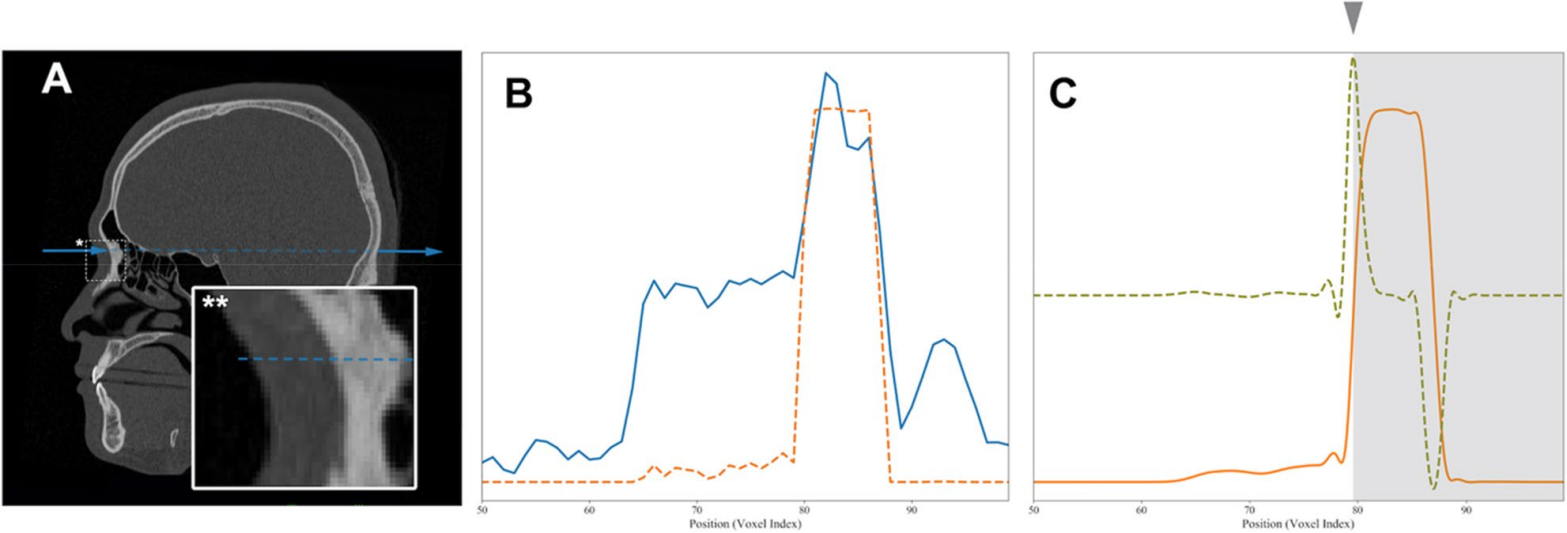
Gradient-based boundary estimation for single-stage DRL. (**A**) A sectional CT image in the sagittal plane with a radiographic beam-mimic line (blue dashed line) passing through a sampled landmark, nasion and surrounding box with asterisk (*). The view of the box region is magnified in the inset box at the left bottom with double asterisks (**) and the y-directional line (blue solid). (**B**) One-dimensional plot of image density profile in Hounsfield units, shown as a blue solid line along the y-directional line passing through the air, through nasion, and the soft and bone tissue, indicated by the blue dashed line in Fig. A. The orange dashed line indicates the bone intensity-enhanced profile. (**C**) Plot of the same source for B showing the non-linear diffusion profile of the bone intensity-enhanced one (orange solid line), its first order derivative profile (light green dashed line), and the final boundary estimation of bone (marked by arrowhead and gray region).

As single-stage DRL is simpler than the multi-stage approach, only two components of 3D coordinates for a landmark could be obtained. The remaining one-dimensional coordinate was inferred by a gradient-based boundary estimation (as shown in the second and third phases of the inferencing stage in Figs. 4 and 5).

The obtained steep gradient changes in CT values at the boundary between the soft tissue and cortical bone (as seen in Fig. 5A) were used to detect the depth of the landmark. If we want to get a landmark on the surface of bone, for example, the nasion point, we first get $x$ and $z$ values of the 3D coordinate by applying the single-stage DRL algorithm on the anterior view of skull. The remaining one-dimensional profile of CT value along the $y$-axis at point $x$ and $z$ can then be obtained by robust boundary detection using the gradient values that we propose here, the bone intensity enhancing function $IE(x)$ being defined as follows:

5 $$IE\left(x\right)=\mathrm{tanh}\left(\frac{x-L}{W}\right)$$

where $x$ is the CT value, $L$ is the center of the CT value range, and $W$ is a scale value. $L$ was 400 and $W$ was 200 for our study. The application of $IE(x)$ turns a one-dimensional profile of CT value (blue line in Fig. 5A) into a simple profile with enhanced bone intensity (orange line of Fig. 5B). The robust calculation of the gradient, however, may suffer from noise contamination. We therefore apply a non-linear diffusion equation using the structure tensor to remove the noise without losing the gradient information^34^. After taking the first order derivative of the noise-reduced profile, the location with maximal gradient is set to the detected bone surface position to determine the remaining coordinate value. Please see Fig. 5C for more details.

### Multi-stage DRL

During our application of multi-stage DRL, the first DRL procedure predicted two coordinate values of a landmark, and these values were used to make the predicted axis for constructing a cutting plane and a new cutaway view. A second DRL was then performed on the newly-constructed cutaway view to calculate the coordinate values again. Most of the landmarks in this study were predicted with excellent accuracy by the first and second DRL, i.e., two-stage DRL, but some landmarks, such as infraorbital foramen, did not yield a satisfactory level of accuracy until the third stage.

Figure 1A–C show sample views of multi-stage DRL with various 3D and cutaway views to determine the right side orbitale point (marked as a light blue point). The $x$ and $z$ coordinate value of right orbitale was predicted by the first DRL on the anterior view of the 3D skull, as shown in Fig. 1A. The sagittal-cut left lateral view was produced for the remaining coordinate value based on the previously determined $x$ coordinate value (Fig. 1B,C); $y$ and $\mathrm{z}$ values were then finally determined by the second DRL agent, as in Fig. 1C.

The prediction of a landmark located inside the skull, such as sella point, was also achieved: two coordinate values of $y$ and $z$ were initially predicted by the first DRL on the median-cutaway left half skull (Fig. 1D,E). This was followed by the construction of another cutaway, based on the previous $\mathrm{z}$ coordinate value, to produce an axial-cut top view (Fig. 1F). Finally, the second DRL could predict $x$ and $y$ coordinate values, as presented in Fig. 1F.

### Implementation

The visualization toolkit was used for the 3D volume rendering^35^. Double DQN implementation is based on the open source framework for landmark detection^25^. The computing environment included Intel Core i9-7900X CPU, 128 GB memory, and Nvidia Titan Xp GPU (12 GB). We set the batch size to 96, discount factor $\gamma$ to 0.9, and experience replay memory size to ${10}^{6}$. We also applied adadelta, an adaptive gradient method for an optimizer. It took approximately 90–120 h to train each individual landmark training model, while inferencing took 0.2 s on average for the landmark detection of a single image view.

## Results

### Landmark localization accuracy

3D coordinate values of landmarks determined by human experts and the experimental values obtained by our proposed method were independently produced and compared in terms of 3D mean distance between them. Details of the results are shown in Table 1; total mean error of the detected landmarks was 1.96 ± 0.78 mm in 3D distance. The detection rate within 2.5 mm of error range was 75.39%, while 95.70% fell within a 4 mm range. The anterior nasal spine point showed the greatest accuracy level with a mean error of 1.03 ± 0.36 mm, the lowest accuracy level occurring at the left porion (2.79 mm).

**Table 1:** Landmark detection rate and three-dimensional mean distance error by landmarks and their types.

Landmarks*		Mean ± SD (mm)	Detection rate (%)			
Name	Type		< 2 mm	< 2.5 mm	< 3 mm	< 4 mm
Bregma	1	1.80 ± 0.65	50.00	87.50	100.00	100.00
Nasion	1	1.71 ± 0.79	65.63	71.88	100.00	100.00
Sella	2	2.39 ± 0.93	37.50	59.38	68.75	100.00
ANS	2	1.03 ± 0.36	100.00	100.00	100.00	100.00
R IOF	2	1.50 ± 0.50	90.63	93.75	100.00	100.00
L IOF	2	2.12 ± 1.29	56.25	56.25	71.88	96.88
R MF	2	1.51 ± 0.55	75.00	96.88	100.00	100.00
L MF	2	1.93 ± 0.55	68.75	87.50	90.63	100.00
R Or	2	1.39 ± 0.47	90.63	100.00	100.00	100.00
L Or	2	1.45 ± 0.39	100.00	100.00	100.00	100.00
R Po	3	1.87 ± 1.08	50.00	59.38	84.38	100.00
L Po	3	2.79 ± 1.14	25.00	50.00	62.50	78.13
R F	3	2.69 ± 0.95	21.88	65.63	68.75	81.25
L F	3	2.68 ± 0.98	18.75	46.88	62.50	90.63
CFM	3	2.09 ± 0.89	53.13	62.50	87.50	96.88
Pog	3	2.35 ± 0.98	40.63	68.75	87.50	87.50
Mean		1.96 ± 0.78	58.99	75.39	86.52	95.70
Type 1^†^	1	1.76 ± 0.55	57.82	79.69	100.00	100.00
Type 2^‡^	2	1.67 ± 0.19	62.89	75.39	84.77	96.88
Type 3^¶^	3	2.41 ± 0.44	54.17	73.96	84.38	92.71

*For all landmarks: Kruskal–Wallis test, p < 0.001.

^†^For all Type1 landmarks: Mann–Whitney test, p = 0.88.

^‡^For all Type2 landmarks: Kruskal–Wallis test, p = 0.01.

¶For all Type3 landmarks: Kruskal–Wallis test, p = 0.56.

For Type 1, 2 and 3 landmarks: two-way ANOVA, p = 0.87. For Type 1 and 3 landmarks: Mann–Whitney test, p = 0.02. For Type 2 and 3 landmarks: Mann–Whitney test, p < 0.0001. For Type 1 and 2 landmarks: Mann–Whitney test, p = 0.92.

*ANS* anterior nasal spine, *IOF* infraorbital foramen, *R* right, *L* left, *MF* mental foramen, *F* mandibular foramen, *CFM* center of foramen magnum. Please refer to Supplementary Table 1 for their definitions and abbreviations.

To determine possible differences in 3D landmark prediction based on the number of DRL passes, we tried four passes of DRL inferencing for each test group landmark. The distance error discrepancies among the repeated predictions at each single- or multiple stage for each landmark ranged from 0 to 0.91 mm, not significantly different (by Friedman test; p > 0.05 for all landmarks; not shown for details).

The prediction error by landmark type was distributed between 1.76 and 2.11 mm in 3D distance (Table 1 and Supplementary Fig. 1). Type 1 and 2 landmarks had similar accuracy, being better than those in type 3; the mean 3D distance error was 1.76 mm for type 1 landmarks, 1.67 mm for type 2, and 2.41 mm for type 3. The statistical differences were not significant among all three types (p = 0.87 by two-way analysis of variance) but were significant between type 1 and 3 (p = 0.02) and type 2 and 3 (p < 0.0001).

**Figure 6:**
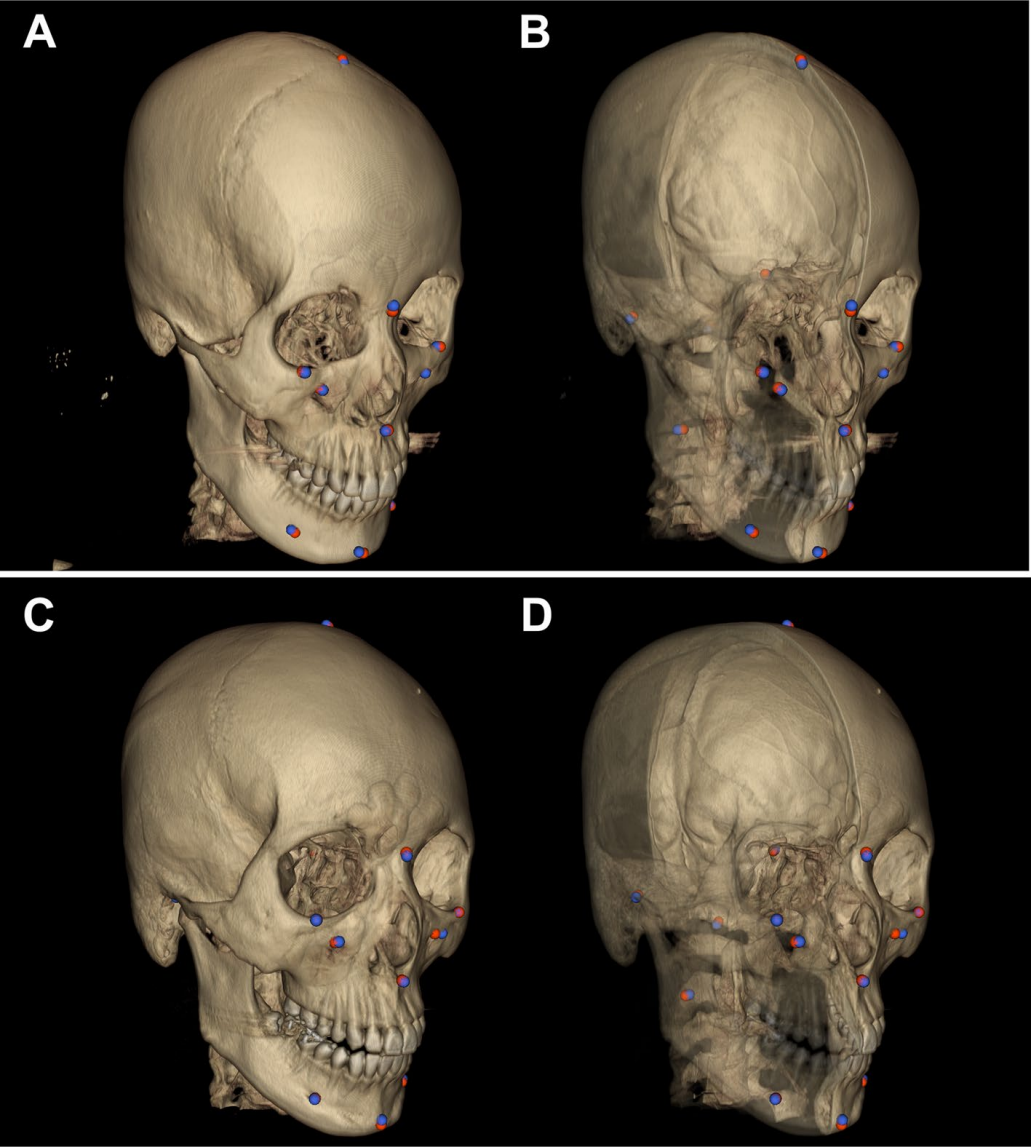
The best and worst landmark prediction results from the test group. **A** and **B** were from Subject 1 (Table 2), which showed the best prediction result of 1.57 ± 0.55 mm in prediction error, while **C** and **D** were from Subject 8, with the worst prediction result (2.41 ± 0.97 mm). The red ball points on and in the skull were produced by the experts and the blue ones by our DRL system. The right half of the skull for each subject is translucent to show the location of predicted landmarks.

We also compared the accuracy levels among the test group subjects, which showed insignificant differences (p = 0.21; Table 2). Table 2 shows that the subject with the best results had 1.57 ± 0.55 mm of prediction error, while the one with the worst had 2.41 ± 0.97 mm. To present this prediction error level visually, the referenced and predicted landmarks for these two subjects are shown with the volume-rendered craniofacial skeletal structures in Fig. 6.

**Table 2:** Three-dimensional mean distance error for the test group subjects.

Subjects	Error distance (mm)	SD (mm)
**Subject 1**	**1.57**	**0.55**
Subject 2	1.58	0.80
Subject 3	1.96	0.61
Subject 4	1.97	1.07
Subject 5	1.98	0.91
Subject 6	1.99	0.86
Subject 7	2.18	0.95
**Subject 8**	**2.41**	**0.97**
mean	1.96	0.84
p*	0.21	

Bold font indicates subjects with the best or worst error level, each of whom is depicted in Fig. 6 with individual landmarks.

*Kruskal–Wallis test.

### Single- versus multi-stage DRL

Most landmarks could be detected by the single-stage approach, except for sella and center of foramen magnum due to their presence in 3D space. The accuracy levels of this single-stage DRL were relatively worse than those of the multi-stage approach. Table 3 compares 3D distance accuracy of single-stage and multi-stage DRL; the mean prediction error using this single-stage DRL was 4.28 ± 3.81 mm, while two-stage DRL yielded 2.04 ± 0.60 mm, and three-stage DRL 1.81 ± 0.43 mm. There were also significant differences among the results of each stage DRL (p < 0.04). The prediction error in single-stage DRL was significantly greater than in multi-stage DRL for most of the landmarks, including anterior nasal spine, porion, mandibular foramen, and infraorbital foramen (p ≤ 0.03). However, the prediction discrepancy of multi-stage DRL for landmarks such as nasion, pogonion, mental foramen, and orbitale was not significantly different from that of single-stage DRL (p ≥ 0.12).

**Table 3:** Variation of landmark detection error by single- and multiple-staged DRL approach (in 3D distance; mm).

Landmark	Type^¶^	Single-stage	Multiple-stage		Cutaway views^‡^	p*
			2 Stages	3 Stages		
Bregma	1	1.80 ± 0.65	–	–	tv	
Sella	2	–	2.39 ± 0.93	–	sclv, actv	
CFM	3	–	2.09 ± 0.89	–	sclv, acbv	
Nasion	1	2.33 ± 1.24	1.71 ± 0.79	–	av, sclv	0.12
ANS	2	4.19 ± 2.98	1.03 ± 0.36	–	lv, av	< 0.0001
Pog	3	3.25 ± 3.11	2.35 ± 0.98	–	av, sclv	0.49
R PO	3	14.22 ± 4.82	1.87 ± 1.08	–	rv, ccpv	< 0.0001
L PO	3	11.56 ± 6.98	2.79 ± 1.14	–	lv, ccpv	< 0.0001
R MF	2	2.64 ± 3.06	1.51 ± 0.55	–	av, actv	0.16
L MF	2	1.82 ± 0.63	1.93 ± 0.55	–	av, actv	0.43
R F	3	4.06 ± 2.10	2.69 ± 0.95	–	scrv, ccpv	0.01
L F	3	3.69 ± 1.62	2.68 ± 0.98	–	sclv, ccpv	0.03
R Or	2	1.25 ± 0.41	1.39 ± 0.47	–	av, sclv	0.13
L Or	2	1.97 ± 1.30	1.45 ± 0.39	–	av, scrv	0.59
R IOF	2	2.58 ± 0.67	1.66 ± 0.62	1.50 ± 0.50	av, sclv, actv	< 0.0001
L IOF	2	4.51 ± 2.12	3.08 ± 1.48	2.12 ± 1.29	av, scrv, actv	< 0.0001
Mean		4.28 ± 3.81	2.04 ± 0.60	1.81 ± 0.43		0.04

¶Landmark types 1, 2, and 3. Please see detailed descriptions in Supplementary Table 1.

^‡^3D and sectional cutaway views: tv (top view), av (anterior view), rv (right view), lv (left view), scrv (sagittal-cut right view), sclv (sagittal-cut left view), actv (axial-cut top view), acbv (axial-cut bottom view), ccpv (coronal-cut posterior view).

* p for 2 stages; by Mann–Whitney test, *p for 3 stages; by Kruskal–Wallis test. p for single-stage vs 2-stage vs. 3-stage; by Kruskal–Wallis test, p = 0.04.

## Discussion

The objective of this study was to develop an automatic 3D cephalometric annotation system by selective application of single- or multi-stage DRL, based on human professional landmarking patterns and characteristics of landmarks. The general scheme of this system is explained in Fig. 4 and can be summarized as follows: 2D image view of the volume-rendered 3D data is first produced to avoid computational burden and complexity. Global feature extraction and selection of 2D cutaway or 3D model view are done. The single- or multi-stage DRL is then implemented to dictate 3D coordinates of target landmarks. The multi-stage DRL is performed by repeated application of single-stage DRL to the various 2D cutaway or 3D views.

Recent 3D automatic cephalometry research poses several challenges in applying machine learning to 3D landmark detection, mainly related to the high dimensionality of the input data. These image data in high dimension incur a high computational cost, a key factor hindering widespread application in clinical, medical, or biological fields. To address this difficulty, several approaches have been utilized: using three orthogonal planes^36^, employing a patch image with RGB color^37^, or extracting a 3D multi-resolution pyramid voxel patch^38^. Kang et al.^27^ recently achieved the image size reduction by down-resampling voxel spacing and applying a convolutional neural network for automatic cephalometry, obtaining about 7.61 mm of error. Ma et al.^39^ reported 3D cephalometric annotation using a patch-based convolutional neural network model with 5.79 mm of mean error. Both these prediction errors seem large and variable from a practical point of view and might be due to the quality of the reduced image or patch acquisition. Because no prediction schemes have been established for objective comparisons of automatic 3D cephalometry (in contrast to 2D cephalometry, for which there exist an open-source database and competition challenges^15,40^), we compared our group's current DRL results with results from our previous non-DRL papers, which used the same radiographic data, landmark definitions, and deep learning methods, to confirm the superior results of DRL (Supplementary Table 3).

Recently, 3D cephalometric studies show accuracy of less than 2 mm of error distance^5,22^. In particular, Lee et al.^22^ produced projected 2D images from a 3D meshed-model and utilized shadowing augmentation to express 3D morphological information on 2D image information. They successfully decreased the prediction error to a mean of 2.01 mm for 7 landmarks; while they tested small numbers in a limited region, one of their 7 landmarks showed an error greater than 4 mm, and the images were produced from the meshed object. Our study tested the landmarks of various regions and achieved both accuracy and stability, as seen in the results. Our more successful results seem related to the stability of the system, the standard deviation of the measurements for all landmarks except two points being less than 1 mm. It should be noted that inter-subject error for the test group was not significantly different and the detection rate, between 2.5 and 4 mm, almost equals that achieved in 2D cephalometry.

In this study, we started with an action pattern analysis of human 3D landmarking for use in implementing automatic cephalometric annotation. Based on our accumulated experience and simple motion analysis of 3D cephalometry, we tentatively concluded that human experts perform 3D landmark annotation sequentially on 3D and multi-planar reconstructed images through multi-step searches as well as a traditional local-to-global approach. This sequential identification-pointing-confirmation procedure can be systematized based on 3D anatomical structural understanding and operator experience. Thus, we assumed that human 3D cephalometric landmark detection is a sequential decision process and can be formulated as a Markov decision process^17^. We here wanted to incorporate a human landmarking-mimic system into our multi-stage DRL system by combining 3D, sectional or cutaway images, their visual direction, and DRL.

Our goal of mimicking human landmarking seems to have succeeded: the sequential selection of view direction with 3D/sectional/cutaway views and DRL application in multiple stages offers good prediction capability. This may be largely due to the efficient 3D point localization offered by DRL. However, the higher error levels, for example, after the right porion (with 14.2 mm of error distance at the initial DRL), clearly suggest that multi-stage DRL led to reduced error levels. In addition, our current DRL system did not implement the full automatic detection process. Further studies will include a more complex decision process which more closely mimics the human decision process for increased accuracy and scalability of DRL.

During the 3D point localization by single- or multi-stage DRL, we wanted to know whether landmark accuracy level could differ depending on anatomical or geometric characteristics. Landmarks are generally classified into three types^28,29^; comparing landmark accuracy levels by type, we found the final mean error, regardless of applied DRL stages, was the greatest in type 3 landmarks (2.41 mm), as compared with those in type 1 and 2 (1.76 and 1.67 mm, respectively). Moreover, the prediction error levels in type 3 landmarks were of greater statistical significance than those of type 1 or 2. Type 3 landmarks include porion, foramen magnum, and mandibular foramen. In the same context, same-stage DRL detection accuracy comparisons yielded similar results. Two-stage DRL was practiced for all landmarks. Though details are not presented here, type 1 landmarks had 1.71 ± 0.79 mm of detection error, type 2 1.72 ± 0.63 mm, and type 3 2.48 ± 1.02 mm. Type 3 landmarks were therefore likely to yield a higher level of detection accuracy at the same stage. We plan to apply multi-stage DRL mainly to type 3 landmarks to increase detection accuracy. We expect that some type 1 and 2 landmarks on the bone surface will achieve good accuracy even using single- or two-stage DRL.

Most 3D cephalometric landmark studies were performed with a segmented or meshed 3D model from 3D CT data after pre-processing^2,4,8,22,41^. We here introduced volume-rendered image modeling instead of mesh-modeling due to the superior speed and quality of modeling. This volume-rendered imaging is also useful in visualizing inner landmarks (located inside the bone coverage), without the additional steps of calculation-meshing-confirmation needed by the meshed-model. Volume rendering can immediately check the object of interest using a cutaway or sectional view by virtue of voxel intensity and ease of transparency processing. This modeling efficiency and qualification can also be applied to the image representation of hole structures, such as foramen or canals, allowing them to be modeled immediately and efficiently, as compared with meshed modeling.

## Conclusion

In this study, we implemented an automatic 3D cephalometric annotation system using single- and multi-stage DRL with volume-rendered imaging based on human sequential landmarking patterns and landmark characteristics. The system mainly involves constructing appropriate 2D cutaway or 3D model views, then implementing a single-stage DRL with gradient-based boundary estimation or a multi-stage DRL to dictate the 3D coordinates of target landmarks. The accuracy using this system clearly suffices for direct clinical applications.

Moreover, our system required no additional steps of segmentation and 3D mesh-object construction for landmark detection. We expect these advantages of our system to enable fast track cephalometric analysis and planning. Future implementations are expected to more closely replicate the human decision process and to achieve greater accuracy through training and testing with larger medical CT datasets.

## Supplementary Information

Supplementary Information.
