# Supplementary material for: 3D cephalometric landmark detection by multiple stage deep reinforcement learning
Source: Sci Rep. 2021 Sep 1;11:17509. doi: 10.1038/s41598-021-97116-7 (PMC8410904; doi:10.1038/s41598-021-97116-7)
Supplement: Supplementary file 1 — Supplementary Information. [file 41598_2021_97116_MOESM1_ESM.docx]

**3D cephalometric landmark detection by multiple stage deep reinforcement learning**

**Sung Ho Kang^1^, Kiwan Jeon^1^, Sang-Hoon Kang^2^, and Sang-Hwy Lee^3,*^**

^1^ Division of Medical Mathematics, National Institute of Mathematical Science, Daejeon, Republic of Korea.
^2^ Department of Oral and Maxillofacial Surgery, National Health Insurance Service Ilsan Hospital, Goyang, Republic of Korea
^3^ Department of Oral and Maxillofacial Surgery, Oral Science Research Center, College of Dentistry, Yonsei University, Seoul, Republic of Korea
^*^[sanghwy@yuhs.ac](mailto:sanghwy@yuhs.ac)

| Landmark | Abbreviation | Bilaterality | Definition |
| --- | --- | --- | --- |
| bregma | Bregma | - | An intersection point of cranial sagittal and coronal sutures joining the parietal and frontal bones come together |
| nasion | Na | - | The most anterior point on frontonasal suture of the midsagittal plane |
| sella turcica | Sella | - | The point representing the midpoint of the pituitary fossa |
| anterior nasal spine | ANS | - | The most anterior point on maxillary bone |
| infraorbital foramen | IOF | Yes | The most superior point at the opening of the maxillary bone located below the infraorbital margin of the orbit. |
| porion | Po | Yes | The most superior point of outline of external auditory meatus |
| mental foramen | MF | Yes | The most anterior point of the small foramen on the anterior surface of the [mandible](https://radiopaedia.org/articles/mandible?lang=us) |
| orbitale | Or | Yes | The most inferior point on margin of orbit |
| mandibular foramen | F | Yes | The entrance point to the [mandibular cana](https://radiopaedia.org/articles/mandibular-canal-1?lang=us)l on the medial surface of the ramus of the [mandible](https://radiopaedia.org/articles/mandible?lang=us) |
| center of foramen magnum | CFM | - | The center point of foramen magnum at the level of basion |
| pogonion | Pog | - | The most projecting median point on the anterior surface of the chin |

**Supplementary Table 1.** Cephalometric landmarks for this study with their definitions and abbreviations.

| **Hyperparameter** | **Value** |
| --- | --- |
| Discount factor, $\gamma$ | 0.9 |
| Batch size | 96 |
| Experience replay buffer size, N | 1,000,000 |
| Target network update frequency, C | 10,000 |
| Learning rate | 0.0002 |
| Initial and final exploration, $\varepsilon$ | 0.9 to 0.1 (linear interpolation) |
| Frames per episode | 1000 |

**Supplementary Table 2.** List of hyperparameters.

unit: mm

|  | Kang (this work) | Yoon (2020) | Kang (2019) |
| --- | --- | --- | --- |
| Algorithm | DRL | Deep learning | Deep learning |
| Bregma | 1.80 ± 0.65 | 1.51 ± 1.23 | 9.37 ± 3.49 |
| Na | 1.71 ± 0.79 | 1.27 ± 0.65 | 7.47 ± 6.17 |
| ANS | 1.03 ± 0.36 | 2.57 ± 1.29 | - |
| R Or | 1.39 ± 0.47 | 2.39 ± 1.18 | 9.57 ± 3.49 |
| L Or | 1.45 ± 0.39 | 2.71 ± 1.04 | 6.66 ± 2.45 |
| R Po | 1.87 ± 1.08 | 2.65 ± 0.60 | 6.37 ± 2.59 |
| L Po | 2.79 ± 1.14 | 1.79 ± 0.84 | 7.05 ± 2.79 |
| R F | 2.69 ± 0.95 | 4.69 ± 3.09 | 8.11 ± 3.79 |
| L F | 2.68 ± 0.98 | 5.06 ± 0.98 | 6.79 ± 3.63 |
| CFM | 2.09 ± 0.89 | 2.32 ± 0.88 | 6.59 ± 2.73 |
| Pog | 2.35 ± 0.98 | 4.76 ± 0.93 | - |
| Mean | 1.96 ± 0.78 | 2.88 ± 1.15 | 7.61 ± 3.61 |

**Supplementary Table 3.** Comparison of accuracy levels in 3D distance by deep learning with and without DRL.


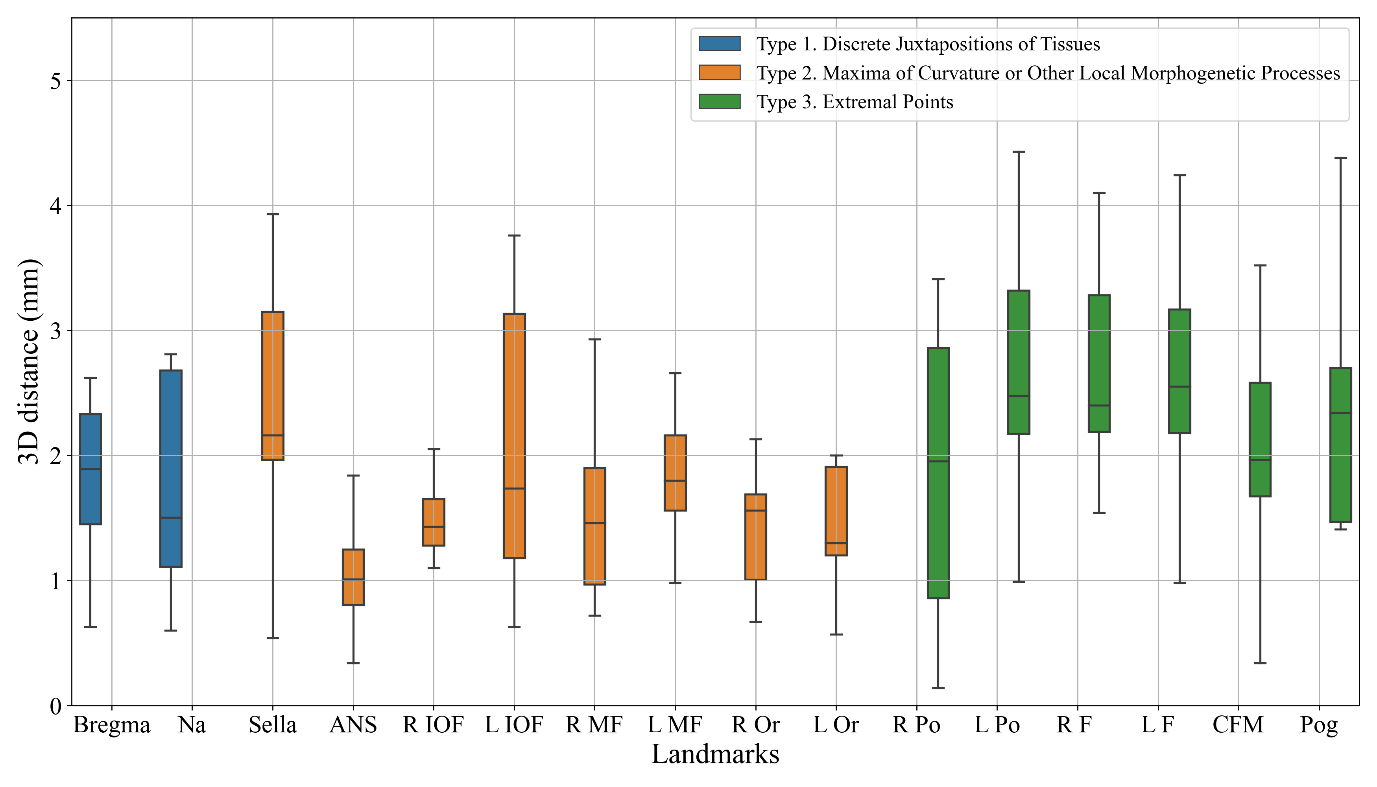


**Supplementary Figure. 1**: Box plot to present the distribution of discrepancy between the predicted and referenced coordinate values of landmarks with type-based colors.

Note 1) Type 1 landmarks (discrete juxtapositions of three structures); blue. Mean discrepancy 1.76 ± 0.72 mm.

Note 2) Type 2 landmarks (on maxima of curvature or other local morphogenetic processes); orange. Mean discrepancy 1.89 ± 0.80 mm

Note 3) Type 3 (endpoints of diameters, centroids, or farthest from segments); green. Mean discrepancy 2.11 ± 0.78 mm


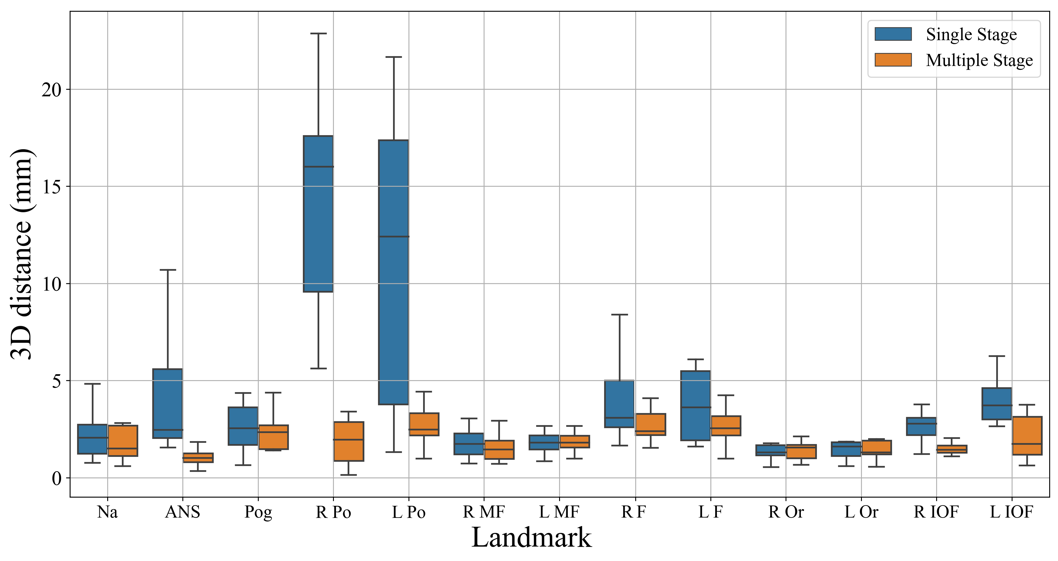


**Supplementary Figure. 2.** Comparison of landmark detection error by single- and multiple-staged DRL. The accuracy level of single- and multi-stage DRL (by mean ± SD) was visualized in boxplot format. The multi-stage DRL error levels (in orange) were smaller in size and range than those of single-stage DRL for all landmarks, except that of right orbitale.
